# Commencement Exercises of the Southern Medical College

**Published:** 1887-04

**Authors:** 


### COMMENCEMENT EXERCISES OF THE SOUTHERN MEDICAL COLLEGE.

The Commencement Exercises of the above Institution took place at the Opera House in Atlanta on the night of the 3d ultimo. There was a very large audience present, composed of the best people of the city. On the rostrum were the members of the Faculty, members of the Board of Trustees, and a number of divines and other distinguished citizens.

After prayer by the Rev. Dr. Martin, Professor Nicolson, Dean of the Faculty, submitted the following report:

To the President and Board of Trustees—At the close of the session of 1886-'7 I have to report a continuation of the remarkable progress that has characterized this Institution since its foundation, eight years ago. Increased facilities for instruction in all the departments of Medicine and Surgery added during the year, especially the large increase of Hospital facilities, now place us upon a plane equal to almost any Institution in this country. The thoroughness of instruction at which we so earnestly aim, has been appreciated by the profession of this and surrounding States, and we shall strive to advance always in this direction. The Senior Class for the session of 1886-'7 numbered forty students, of whom we present this evening thirty as having passed satisfactory examinations, and recommend that they receive the degree of Doctor of Medicine.

The diplomas were then presented by the Dean. Each graduate came forward, as his name was called, and received his diploma from the hands of Professor Nicolson. Among the members of the graduating class were J. E. Kintz, of Ohio, chairman of the Committee of Invitation, one of the brightest and best of the students, and also Clifford A. Stiles, of Atlanta, endeared to every heart, whose deaths the Class had recently been called upon to mourn. As a most appropriate and touching tribute and memento, the diploma to which each would have been entitled was draped in emblems of mourning and presented to their respective friends. It was announced that John A. Ison, of Alabama, was absent on account of sickness.

After the diplomas had been distributed, the President of the College, Dr. Thomas C. Powell, addressed the graduates, saying :

Young Gentlemen—By virtue of the authority conferred upon me by the Legislature of Georgia and the Board of Trustees of the Southern Medical College, and also by the fact that, on examination, you have been found to have fulfilled the requirements, according to the rules of our Institution, I confer upon each of you the Degree of Doctor of Medicine, with all of the rights and privileges pertaining thereto.

The following are the names of the graduates :

W. H. Aycock, Georgia.
James Bailey, Texas.
H. C. Bates, Georgia.
B. W. Bizzell, Alabama.
N. H. Boland, Georgia.
A. S. Bridwell, Georgia.
A. O. Brooks, Georgia.
M. M. Crowder, Georgia.
C. M. Curtis, Georgia.
J. A. Dobbins, South Carolina.
B. W. Fite, Georgia.
W. L. Green, Georgia.
J. A. Ison, Alabama.
G. W. Julian, Georgia.
J. C. Kerr, Tennessee.
M. H. Lee, Tennessee.
T. D. McDaniel, Georgia.
W. A. Means, Georgia.
J. E. Miller, North Carolina.
S. R. Mitchell, Georgia.
H. A. Mobley, Georgia.
A. J. Morris, Alabama.
C. T. Quinn, Georgia.
J. R. Reeve, Texas.
D. D. Reid, Georgia.
J. T. Renouff, Connecticut.
W. R. Rice, Georgia.
T. B. Robertson, Alabama.
D. M. Wheelis, Alabama.
C. M. Wyatt, South Carolina.

Having conferred the degree, President Powell addressed the graduates as follows :

Young Gentlemen of the Profession—I am pleased to say to you that, in accordance with the laws of Georgia, you are now Doctors of Medicine. It is important you should feel impressed with the full significance of that title before you can have a true conception of the real object of medical science and practice, and what should be a physician's character, attainments and usefulness.

I am glad to know that the signs of the times indicate a revolution in public thought as regards character in individuals, professions, finances, labor, and all social and public questions. The corruption that has been insinuating itself into the body politic, and, consequently, into social circles and every department of business, is at last arousing all true men and women to its enormity and its destructive tendencies. Reform is beginning to be the demand, and with no uncertain sound, on every hand ; and if individuals, and all bodies of men, do not voluntarily drop the curtain behind which they have entrenched unrighteous principles and practices, the public itself, as it becomes more and more enlightened, will tear away the veil and reveal the hideous *mokanna* behind its folds.

Public thought, like History, moves in cycles, and the period of the cycle that is resonant with voices calling for purer methods and higher attainments in all things, is now moving around just in front of us.

Let me urge you, young gentlemen, at the beginning of your professional career to fall into that line, and, with high resolves and noble efforts, keep step to the music of progressional reform. The man who first steps into this line will win the high position of a bold and righteous leader of pure, exalted public thought. He will not wait, as so many men do, to reflect the opinions of others, but he will mold opinions for himself out of the eternal words of Truth, and thus panoplied in the grandeur of moral heroism, he will not be afraid to avow them to the world.

In no utterance of his had Solomon shown himself the wisest of men than when he said a good name was to be chosen rather than great riches. Next to life, your character is the most precious thing you can possess this side of the grave ; yet character cannot be bought with silver or gold ; it cannot be inherited, nor be created by any external advantages ; it is built by the inner man and by invisible hands. Hence it can be attained by the poor as well as the rich. Unlike reputation, it has no need to depend upon the shifting winds of popular opinion. It erects its own sublime and enduring fabric. Its architect is a noble and fearless spirit that slowly and grandly builds the structure out of the priceless materials of Truth, Virtue and undeviating Integrity, and crowns it all with the capstone of HONOR. Then, decked with the ornaments of Charity, Kindness, and Benevolence, the three linked jewels of man's love to man, it needs no insignia of rank save its own patent of nobility of the soul.

Slander may sometimes assail a spotless man, but its missels of hate eventually fall to the ground. A good character is in itself a protection from suspicion and evil reports, and no power but that of its possessor can prevent it from standing intact in time and eternity. One of the first requisites, my young brethren, for success in your career is to have an intelligent and comprehensive knowledge of your work, and the next should be a worthy ambition in regard to that work. The profession of medicine gives a wide and peculiar scope to the incitement and fulfillment of many of the noblest aspirations with which the mind can be inspired. But if your ambitions are not honorable, neither will be your achievements ; and, whether personally or professionally, it is a grand thing to have that glorious title " a man of honor."

Great action in any career is always preceded by a great purpose. After having marked out your course, the next thing is a manly determination to pursue it to the end. To do this you will need, first, a profound sense of DUTY, that crowning gem of all principles. A resolve " to dare nobly, to will strongly, and never to falter in the path of duty," will make a man stand to his post and die there if necessary, and when success comes to him it will be without a stain, and his fame without a flaw.

The faithful discharge of duty will also inspire you with courage, perseverence and energy—all of them very important elements of success. But do not confound energy with mere activity, or with rashness. It has been truly said " Energy is a Bucephalus, guided by the hand of an Alexander ; rashness is a Mazeppa's fiery steed, unbridled and unrestrained, bearing its rider over hill and dale to probable destruction. The former is power, guided by wisdom ; the latter is power, goaded to action by blind impulse." In the meantime, do not be impatient of results, but cherish only the noble discontent which aspires after better and greater things than you have yet accomplished. The final triumph of all science and all labor has been made by "the patient persevering process of accretion, which builds the ant-heap, particle by particle, thought by thought, fact by fact." The grandest results cannot be attained in a day, and all progress of the best kind is gradual.

Do not forget, young brethren, that your career is just begun, and that while it is important that you should continue to study and increase in the knowledge of all that pertains to your profession, there is other information to be obtained in order to furnish the needful requirements for a higher position in the medical profession. The general information of a Doctor of Medicine can scarcely be too broad. He should have a wide and deep knowledge of men, and of the affairs which interest all classes of men, including literature, art, science, political economy, religion, labor, trade, etc.

It should be the aim of the young professional to seek the company of the wise, the intelligent, and the good, not only for the influence upon his own moral character but also upon his mental strength and growth. The character of your associates will be regarded as your own. There is nothing truer than the old adage: "Show me with whom thou goest and I will tell thee what thou dost." Bad qualities in men are catching, as well as diseases, and the mind is as much, if not a great deal more, liable to infections than the body. So it is better and wiser to keep company with persons rather above than below yourself, and choose for an associate a spirit similar, yet superior, to your own. Mental and spirtual superiority are also the inspirers of true politeness, another attainment very essential to a professional character and career.

The true gentleman, in his business as well as in society, is recognized by his scrupulous regard for the rights and feelings of others, even in trivial matters. Therefore, true politeness has no classification, and the rich and the poor alike share its justice and humanity. Some one has beautifully said, that Humanity knows itself to be a king, though dethroned and crownless, and it will be treated with respect; therefore true politeness is the glory of any young man, as well as being a great aid to success in his life work.

The rules of medical ethics, which should govern the attitude of physicians towards each other, and their patients, are in keeping with what should be the high character of the profession. A disregard of this code will make you unworthy of your vocation, and divest you of the character of a man of honor. If a physician, on account of the illness of himself, or from other causes, should be compelled to depend upon one of you to attend his patients, though your compliance is an act of professional courtesy, still it should be given with a due regard to the position of the physician regularly employed in the family. If he returns from any enforced absence, you are expected, and it is your duty, to turn over the cases to him and the fees accruing from medical attendance. Surgical or other difficult cases are exceptions. These fees should be given to those who officiate. While thus obliging a medical brother, it would be dishonorable to employ the cunning of policy or any other underhanded means or measures to usurp his position in the family as its regular physician. The welfare of the patient should be your only object in consultations with medical men. The family physician is expected to have the preference in giving his views of the case at the first or subsequent consultations. These meetings should be characterized by a tone of candor. confidence and fraternal courtesy, rather than a spirit of rivalry or jealousy, while each one considers and acts for the sole good of the patient. In this, as in all other things, be guided by that noblest and grandest maxim ever written in any code: "Do unto all men as you would that they should do unto you." Let that maxim be the mentor and guide of your lives, young gentlemen; and, whether you reach the lofty pinnacle of fame in medical science or your work is only nobly done among the lowly and obscure, you will reap your reward, and honor and success will crown your career. I am glad to know that, in the present and increasing revolution of public thought, if a man, as a Doctor of Medicine or of any other avocation, can truly lay claim to that proudest of all titles, "a Christian Gentleman," it is at once a passport to what is usually the best society, and enables him to deservedly win the confidence of friends, patrons, and the public

Young gentlemen, let your first aspiration, and your greatest endeavors, be to engrave this title upon the escutcheon of your manhood. Resolve, from this hour, that you will set for yourself a high ambition. Your duties afford you a great and glorious field. The race to usefulness and greatness is before you. You should oppose everything that detracts from so fair a prospect. Determine to excel in all that is good and noble, and success will crown your efforts. The good, the true, the sublime will be always around you. You will be blessed with sunshine and flowers, crimson clouds and rainbows, bright homes, warm and genial hearts to bind you to life and point you to Heaven, the perfection of the Sublime and Beautiful!

Now, let me entreat you to fully appreciate and truly estimate the grandieur of your profession. Work, toil, and struggle to place it before the world in its true, commanding and heavenly light. Consecrate your life to usefulness and honor, and you will always have sunlight to paint an iris over "smiles and tears" and your labor, in God's own good time, will yield a golden harvest. And may I not hope that, when for all of us "Life's fitful fever is over," I will clasp your hands among that "goodly company" which will assemble by the River of Life, and never be dissolved, as the blessed and eternal ages roll on?

The able and eloquent address of Professor Powell was admirably delivered, and commanded the profound attention of the Class and of the entire audience.

Rev. Dr. Quigg, of Conyers, Ga., the orator selected for the occasion, was then introduced, and, in the language of our city papers, "made a magnificent address." His subject, "The obligations which rest upon the Medical Practitioner," was handled in a most masterly manner. Dr. Quigg spoke in glowing terms of the rapid rise of the Southern Medical College, of the high character and acknowledged ability of the Faculty, and of the fitness of Atlanta for a great medical school of the South. He passed a high eulogy upon the Institution for what he considered a new and most worthy departure—namely, the inculcation of morals in connection with medical instruction. The important place of the physician in the community, and his influence for good or evil, was impressed in terms which evidently went home to the hearts of the graduates; and the entire address was delivered with that full melodious voice, strong language, rich Irish fervor, and impassioned eloquence for which Dr. Quigg is noted.

The Valedictory Address, by a member of the Class, Dr. A. S. Bridwell, was then delivered. The young man acquitted himself with much credit. His address was well conceived, couched in good and appropriate language, gracefully delivered, and had the rare virtue—brevity.

4

The Prizes were next delivered, in a very appropriate and pleasing manner, by Captain Burke, of the Gate City Guards, as follows:

First Honor, by the Faculty, to Dr. B. W. Bizzell.

Second Honor, to Drs. H. A. Mobley and J. T. Renouff, who received a tie vote.

The individual Prizes of the Professors were awarded as follows:

For best examination—

On Obstetrics, by Professor Powell, to Dr. H. C. Bates.

In Surgery, by Professor Gaston, to Dr. A. O. Brooks.

In Physiology, by Professor Word, to Dr. W. H. Aycock.

In Anatomy, by Professor Nicolson, to Dr. C. N. Wyatt.

In Eye and Ear, by Professor Hobbs, to Dr. C. F. Quinn.

In Materia Medica, by Professor Roy, to Dr. J. A. Ison.

In Practice of Medicine, by Professor Bizzell, to Dr. C. M. Curtis.

In Chemistry, by Professor Burns, to Dr. S. R. Mitchell.

A Premium, by Mr. M. C. Kizer, for the best Essay, was awarded to Dr. B. W. Bizzell.

The several parts of the programme were interspersed with delightful music. Numerous ladies in the audience sent up their floral offerings to the young and happy graduates, and the entire occasion was among the most attractive, instructive and pleasant that has ever occurred in the city.

